# The effects of uninterrupted and interrupted sitting on blood pressure and arterial stiffness in patients with established coronary heart disease

**DOI:** 10.1113/EP093399

**Published:** 2026-03-31

**Authors:** Simon Fryer, Eve Scarle, Louise Turner, Arsalan Moinuddin, James Faulkner, Hayley Legg, Craig Paterson, Keeron Stone

**Affiliations:** ^1^ School of Health, Education, and Science, Oxstalls Campus University of Gloucestershire Gloucestershire UK; ^2^ Primary Care Research Centre, Aldermoor Health Centre University of Southampton Southampton UK; ^3^ Population Health Sciences, Bristol Medical School University of Bristol Bristol UK; ^4^ Centre for Cardiovascular Research, Innovation and Development (CURIAD) Cardiff Metropolitan University Cardiff UK; ^5^ National Cardiovascular Research Network Cardiff UK; ^6^ Shri Guru Ram Rai Institute of Medical & Health Sciences Dehradun India

**Keywords:** cardiac rehabilitation, cardiovascular disease, endothelial function, heart disease, lifestyle behaviours, prolonged sitting

## Abstract

Sedentary behaviour is an independent risk factor for cardiovascular disease. In healthy adults, prolonged uninterrupted sitting acutely increases blood pressure (BP) and aortic stiffness; however, these effects can be mitigated with light physical activity interruptions. Whether such mitigation strategies are effective in at‐risk populations remains unclear. This study examined the effects of uninterrupted and interrupted sitting on BP and arterial stiffness, measured by carotid‐femoral pulse wave velocity (cfPWV), and femoral‐ankle PWV in patients with established coronary heart disease (CHD). Using a randomised cross‐over design, 14 CHD patients sat for 2 h uninterrupted (control [CON]), and 2 h interrupted with light physical activity (sit‐to‐stand, calf raises and walking) breaks every 30 min (ACT). Brachial BP and cfPWV were assessed immediately pre‐ and post‐sitting. Time‐by‐condition effects were tested using linear mixed effects models with baseline adjustments. A significant time × condition interaction effect was detected for systolic BP (*P* = 0.037) with an increase in CON (mean difference [MD] = 15 mmHg [95% CI: 8, 23], *P* < 0.001) but not ACT (MD = 4 mmHg [95% CI: −4, 11], *P* = 0.334). A significant time effect was detected for cfPWV, with an increase across both CON and ACT conditions (MD = 0.76 m/s [95% CI: 0.52, 0.99], *P* < 0.001). For CHD patients, light activity breaks every 30 min can attenuate the impact of prolonged sitting on BP but not arterial stiffness; higher frequency or intensity of activity breaks may be required for better preservation of cardiovascular function.

## Abstract (highlights)

- **What is the central question of this study?**
  Do simple light physical activity interruptions attenuate the detrimental responses to 2 h prolonged sitting on blood pressure and arterial stiffness in a population with established coronary heart disease?
- **What is the main finding and its importance?**
  Light physical activity interruptions appear sufficient to reduce blood pressure in patients with established coronary heart disease. However, higher intensity, more frequent interruptions may be necessary to fully attenuate its impact on arterial stiffness.

## 1 INTRODUCTION

Sedentary behaviours, defined as being in a seated, reclined or lying posture with a low energy expenditure (≤1.5 METS) (Tremblay et al., 2017), have been found to be an independent risk factor for cardiovascular disease (CVD) (Ekelund et al., 2016). Recent meta‐analyses have shown that uninterrupted sitting elevates systolic blood pressure (SBP) by ∼3.2 mmHg, whereas introducing activity breaks reduces this by ∼4.4 mmHg (Paterson et al., 2021). However, much of the previous research has focused upon young, healthy populations (Adams et al., 2024; Fryer et al., 2021; Weston et al., 2019) with little attention paid to groups at a greater risk of sitting‐induced cardiovascular dysfunction, where there may be a greater propensity for a worse response to uninterrupted prolonged sitting, and therefore known physical activity strategies may not be effective.

In older hypertensive adults (62 ± 6 years) with type 2 diabetes, Dempsey et al. (2016) reported that 8 h of uninterrupted sitting increased SBP by 10 mmHg and diastolic blood pressure (DBP) by 5 mmHg. These increases exceeded the changes reported in young healthy adults and are above the 5 mmHg threshold considered to be a clinically significant change (Dempsey et al., 2016; Rahimi et al., 2021). Fortunately, these increases in blood pressure (BP) were significantly reduced when light walking was used to interrupt sitting. While informative, the interpretability/applicability of these findings is limited due to the 8 h sitting exposure, which far exceeds the typical free‐living maximum of 2 h uninterrupted sitting (O'Brien et al., 2022). In addition, we do not know what may happen in people who have other diseases known to impair cardiovascular function, such as older adults with established coronary heart disease (CHD). Given that CHD is characterised by widespread endothelial impairment and a reduction in nitric oxide bioavailability, and is associated with advanced vascular remodelling and aortic stiffening (Matsuzawa & Lerman, 2014), the current and established physical activity interruption strategies (Paterson et al., 2021) may not be effective in this population. For example, previous research has shown that exercise‐based cardiac rehabilitation does not always translate to a reduction in BP (Sahin et al., 2021). As such, we need to identify whether physical activity interruptions to prolonged sitting are effective in a population with CHD, or whether they need specific approaches to help reduce the sitting‐induced cardiovascular burden. This will be of use when informing future randomised control trials, as well as sedentary behaviour guidelines for at‐risk populations.

The aim of this study was to determine whether simple light physical activity interruptions can attenuate the expected detrimental responses to 2 h prolonged sitting on cardiovascular function in a population with established CHD.

## 2 METHOD

### 2.1 Ethical approval

Prior to recruitment and data collection, institutional ethical approval from the University of Gloucestershire, UK, was obtained (REC.21.53.3), which conformed to both the standards of the journal, as well as the *Declaration of Helsinki* 2024, except for registration in a database (clause 35). All participants gave written informed consent.

### 2.2 Participants

This study is reported in accordance with the Consolidated Standards of Reporting Trials guidelines (Schulz et al., 2010). Fourteen male participants with established CHD (characteristics in Table 1) were recruited. All participants were asymptomatic of any acute illness, and were physically active, engaging in a minimum of two Phase Four cardiac rehabilitation classes per week. Phase Four cardiac rehabilitation consists of a long‐term community‐based exercise and education programme run by specially trained instructors. All participants were non‐smokers and on medications listed within Table 1.

**TABLE 1:** Participant characteristics and medication use.

	Value
Characteristic (mean (SD))	
Age (years)	78.4 (6.24)
Height (m)	1.78 (0.04)
Weight (kg)	81.7 (11.2)
BMI (kg/m^2^)	25.5 (3.7)
Medication use (*n*)	
Aspirin	8
Amitriptyline	2
Levothyroxine sodium	4
Perindopril	5
Rosuvastatin	8
Bisoprolol	4
Eliquis	6
Atorvastatin	9
Ramipril	3
Omeprazole	7

Abbreviations: BMI, body mass index; *n*, number of participants.

### 2.3 Experimental protocol

The experimental protocol consisted of three separate visits to a laboratory within a 10‐day period. During visit 1, participants gave their consent before having their height, body mass and health status assessed using a physical activity readiness questionnaire. Participants were then familiarised with all experimental procedures and equipment. The two following visits consisted of an uninterrupted condition (Control [CON]), and an interrupted 2 h sitting condition (Activity [ACT]), of which the order was block randomised in Microsoft Excel. Participants were randomised in blocks of seven to one of two treatment sequences (CON, ACT). The resulting order of conditions was not revealed to the participant until the morning of their first condition to avoid any conscious or unconscious bias towards a particular condition. Each session commenced between 08.30 and 10.00 h following a minimum 4 h fast, consuming only water and having refrained from strenuous exercise and alcohol for a 24 h period. An overnight fast was not used as participants needed to take medication in the morning, often with a small meal. For each participant, the start time remained consistent for each condition.

At the start of each experimental visit, participants were asked to empty their bladder and bowel before quietly lying supine on a test bed for 10 min. During this period, participants were fitted with all equipment, including an oscillometric blood pressure cuff (SphygmoCor Xcel, Atcor Medical, Sydney, Australia) over the upper left arm to determine all pulse wave analysis (PWA) variables. To determine carotid‐femoral pulse wave velocity (cfPWV) and femoral‐ankle pulse wave velocity (faPWV), pressure cuffs were placed over the left thigh and ankle, respectively. The aortic‐femoral stiffness gradient (af‐SG) was subsequently calculated off‐line. To estimate changes in venous pooling over the 2 h sitting protocols, pre‐ and post‐calf circumference was assessed. After all baseline assessments were completed, the participant was moved into a seated position on a comfortable armchair for 2 h. Following the 2 h sitting period, participants had their calf circumference reassessed before being asked to lie supine for 10 min while all baseline assessments were repeated in accordance with.

### 2.4 Experimental procedures

#### 2.4.1 Physical activity interruption

The physical activity interruption lasted approximately 5 min and was conducted by all participants at the time points 30, 60 and 90 min after the start of sitting. The activity involved 5× stand to sits from their chair, 5× upright wall supported calf raises and 3 min of gentle walking. The choice of activity was designed to be feasible and mimic that which could likely be done at home during television advert breaks or breaks from reading.

#### 2.4.2 Pulse wave analysis

The SphygmoCor Xcel was used to conduct pulse wave analyses (PWA) pre‐ and post‐sitting. In brief, oscillometric pressure waveforms are assessed during a brachial cuff inflation lasting approximately 30 s, this is followed by a 10 s sub‐diastolic recording, of which a corresponding aortic waveform is generated using a validated transfer function (Butlin et al., 2012). From sub‐diastolic recording, central: systolic blood pressure (cSBP), pulse pressure (cPP), augmentation index (AIx), and augmentation index normalised to a heart rate (HR) of 75 beats/min (AIx@75) were derived.

#### 2.4.3 Pulse wave velocity

The SpygmoCor XCel device was used for all PWV analyses as it enables simultaneous assessment of proximal and distal arterial waveforms using a tonometer and volume displacement cuff. PWV is calculated by dividing the arterial path length, or PWV distance (*D*), by the arterial pulse transit time (PTT). For cfPWV, the tonometer was placed on the right carotid artery and the oscillometric cuff was placed on the right thigh at the level of the femoral artery, following the manufacturer's guidelines (Butlin et al., 2013). Using large Seca callipers (Seca 207, Seca, Birmingham, UK), the carotid‐femoral *D* was estimated by measuring the linear distance from the suprasternal notch to the top of the cuff at the centre line of the leg and subtracting the distance from the suprasternal notch to the carotid artery. For faPWV, the tonometer was placed at the point of maximal pulsation (obtained by palpation) at the level of the superficial femoral artery, whilst the ankle cuff (SC10, Hokanson, WA, USA) was positioned with the bottom edge proximal to the malleolus. Femoral‐ankle *D* was estimated by measuring the linear distance from the point of tonometric applanation to the top of the ankle cuff at the centre line of the leg with large Seca callipers. Femoral‐ankle PTT was corrected prior to the calculation of PWV as previously described (Stone et al., 2019). The afSG, which we have shown to have good between‐day reliability (Stone et al., 2021b), was calculated as a ratio of lower‐limb PWV to central (aortic) PWV in accordance with Stone et al. (2021a), as this has been shown to be an enhanced independent CVD risk assessment tool for older adults (Stone et al., 2024).

#### 2.4.4 Sample size

Sample size calculation was based on sitting‐induced changes in mean arterial pressure (MAP). Using the pooled result from previous studies conducted in an equivalent population as the effect size of interest (standardised mean difference [SMD] = 0.40) (Paterson et al., 2021), an α‐level of 0.05, a β of 0.8 and an assumed correlation between measurements of 0.83 (Balk et al., 2013), it was estimated that 14 participants would be required to detect an effect.

### 2.5 Statistical analysis

**FIGURE 1:**
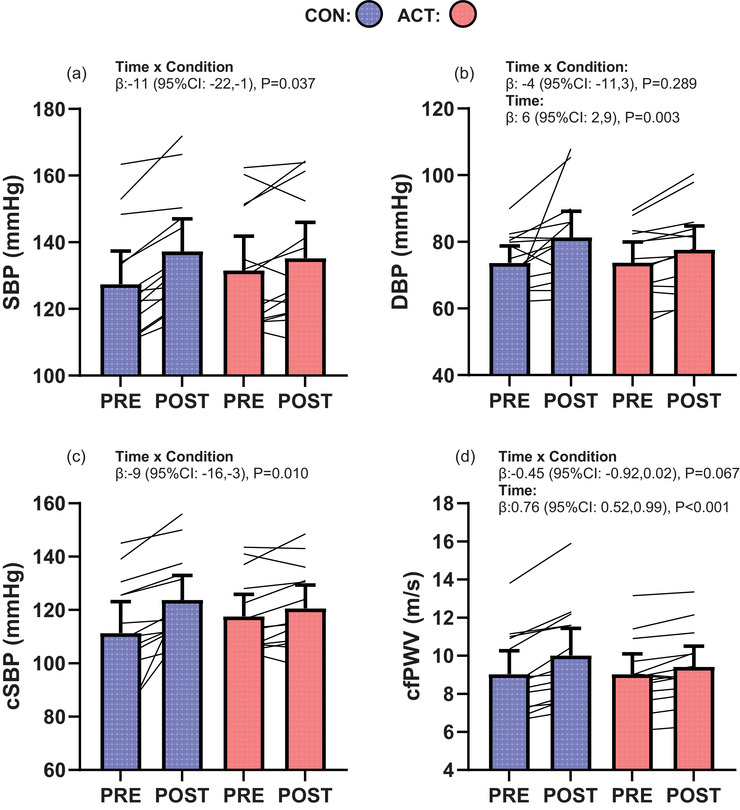
Systolic blood pressure (a), diastolic blood pressure (b), central systolic blood pressure (c) and carotid‐femoral pulse wave velocity (d) responses to 2 h (pre vs. post) uninterrupted sitting (CON) and 2 h sitting interrupted with light physical activity (ACT). Data presented (all *n* = 14, except cfPWV *n* = 13) are means and 95% CIs and are derived from baseline‐adjusted mixed models. PWA data reported without decimal places (DP), PWV data reported to 2 DP due to the meaningfulness of small changes. cfPWV, carotid to femoral pulse wave velocity; cSBP, central systolic blood pressure; SBP, systolic blood pressure.

All statistical analyses were conducted using Jamovi (Version 1.8), a graphical front end to the R programming language. All data were normally distributed. There were no missing data except for cfPWV, for which one participant's carotid waveform acquisition was of insufficient quality to generate a reliable measurement. As such, analyses involving cfPWV and afSG were conducted with a sample size of *n* = 13. The general analysis for linear mixed models (GAMLj) module (Jamovi) was used to assess changes in all BP and arterial stiffness variables. In each model, the pre‐sit data for that outcome from each condition were used as a covariate to account for between condition variability. Given the interdependence of BP and arterial stiffness, MAP was used as a covariate for cfPWV and faPWV analyses. Where a significant interaction effect was reported, the simple effects estimate with 95% confidence intervals [CI] are reported for the pre versus post change in each condition. Where a significant time effect was reported, estimated marginal means with standard error (SE) were used to determine the pre (0 min) versus post (2 h) difference of the combined uninterrupted and interrupted conditions. For everything else, raw data are presented as means ± standard deviation (SD), and mean difference (MD) with 95% CI. Data in Figure 1 are presented as mean and 95% CIs derived from the unadjusted models. The level of significance for all analyses was set at α = 0.05.

## 3 RESULTS

### 3.1 Participant characteristics and medication

Participant characteristics and medication are shown in Table 1.

### 3.2 Blood pressure

Following adjustment for baseline values, Figure 1 and Table 2 show a significant interaction effect for SBP and cSBP only. Simple effects revealed that SBP significantly increased in the CON condition (*P* < 0.001; MD = 15 mmHg, 95% CI: 8, 23) but not the ACT condition (*P* = 0.334; MD = 4 mmHg, 95% CI: −4, 11). cSBP significantly increased in the CON condition (*P* = 0.001; MD = 12 mmHg, 95% CI: 8, 17) but not the ACT condition (*P* = 0.222; MD = 3 mmHg, 95% CI: −2, 8). There was a significant time effect for MAP, HR, DBP, AIx and AIx@75. The estimated marginal means revealed that MAP significantly increased, from 90 mmHg (SE = 1) to 92 (SE = 1), as did DBP, from 74 mmHg (SE = 1) to 80 mmHg (SE = 1). HR significantly decreased over the 2 h from 57 beats/min (SE = 0.6) to 55 (SE = 0.6). AIx and AIx@75 decreased over the 2 h from 28 (SE = 1) to 20 (SE = 1), and from 19 (SE = 1) to 11 (SE = 1), respectively.

**TABLE 2:** Raw mean (SD) central and peripheral blood pressure data pre and post 2 h of uninterrupted (CON) and interrupted (ACT) sitting (*n* = 14).

	MAP (mmHg)	SBP (mmHg)	cSBP (mmHg)	DBP (mmHg)	cPP (mmHg)	HR (beats/min)	AIx (%)	AIx@75 (%)	PF (mmHg)	PB (mmHg)	RM%
CON											
0 min	89 (11)	122 (27)	111 (20)	74 (10)	43 (10)	55 (11)	28 (10)	18 (12)	27 (7)	17 (4)	64 (8)
2 h	92 (14)	137 (17)	124 (14)	81 (14)	45 (26)	54 (11)	21 (11)	11 (13)	27 (7)	18 (5)	65 (9)
ACT											
0 min	91 (12)	132 (18)	118 (15)	74 (11)	45 (9)	59 (12)	28 (9)	20 (14)	29 (8)	18 (4)	64 (12)
2 h	92 (13)	135 (19)	121 (15)	78 (12)	52 (9)	57 (14)	19 (9)	11 (13)	29 (6)	18 (4)	61 (11)
Interaction effect											
*P*	0.226	0.037*	0.010*	0.289	0.106	0.788	0.569	0.493	0.907	0.472	0.241
β (95% CI)	−3 (−7, 2)	−11 (−22, −1)	−9 (−16, −3)	−4 (−11, 3)	−9 (−20, 2)	−0.4 (−3, 3)	−2 (−9, 5)	−3 (−9, 4)	−0.3 (−5, 4)	−0.9 (−4, 2)	−3 (−9, 2)
Time effect											
*P*	**0.037** ^†^	**<0.001** ^†^	**<0.001** ^†^	**0.003** ^†^	0.106	**0.007** ^†^	**<0.001** ^†^	**<0.001** ^†^	0.700	0.931	0.324
β (95% CI)	2 (0, 4)	8 (4, 11)	8 (4, 11)	6 (2, 9)	5 (−1, 10)	−2 (−4, −1)	−8 (−11, −4)	−8 (−11, −5)	0.4 (−2, 3)	0.1 (−1, 1)	−1 (−4, 1)
Condition effect											
*P*	0.206	0.180	**0.036** ^#^	0.290	0.077	0.836	0.597	0.612	0.133	0.508	0.270
β (95% CI)	−1 (−4, 1)	−4 (−7, −0)	−4 (−7, −1)	−2 (−6, 2)	−5 (−10, 1)	−0.1 (−2, 1)	−1 (−4, 3)	−1 (−4, 3)	2 (−0.5, 4)	−0.9 (−4, 2)	−3 (−9, 2)

*Note*: Data are means (SD). PWA data reported without decimal places for meaningfulness. ^†^Significant time effect; ^#^significant condition effect; *significant interaction effect. Abbreviations: AIx, augmentation index; AIx@75, augmentation index normalised to a heart rate of 75 beats/min; cPP, central pulse pressure; cSBP, central systolic blood pressure; MAP, mean arterial pressure; PB, pressure backwards; PF, pressure forwards; RM%, reflection magnitude percentage; SBP, systolic blood pressure.

### 3.3 Pulse wave velocity

Table 3 shows there were no significant interactions, or condition effects for any PWV variables. There was a significant time effect for both cfPWV and the af‐SG, with no change in faPWV. The estimated marginal means (SE) for cf‐PWV and af‐SG revealed an increase from 9.06 (0.42) to 9.63 (0.42) m/s, and a decrease of 1.31 (0.07) to 1.24 (0.07), respectively.

**TABLE 3:** mean (SD) arterial stiffness data pre and post 2 h of uninterrupted (CON) and interrupted (ACT) sitting.

	cfPWV (m/s)	faPWV (m/s)	af‐SG
CON			
0 min	9.03 (2.12)	11.00 (0.85)	1.30 (0.29)
2 h	10.00 (2.46)	11.5 (1.16)	1.22 (0.29)
ACT			
0 min	9.04 (1.84)	11.4 (1.02)	1.33 (0.27)
2 h	9.42 (1.87)	11.4 (0.96)	1.27 (0.24)
Interaction effect			
*P*	0.067	0.098	0.588
β (95% CI)	−0.45 (−0.93, 0.02)	−0.52 (−1.12, 0.10)	0.02 (−0.06, 0.10)
Time effect			
*P*	**0.001^†^**	0.110	**0.002^†^**
β (95% CI)	0.76 (0.52, 0.99)	0.25 (0.10, 0.60)	−0.07 (−0.11, −0.03)
Condition effect			
*P*	0.066	0.202	0.597
β (95% CI)	−0.23 (−0.46, 0.01)	−0.20 (−0.50, 0.10)	0.01 (−0.03, 0.05)

*Note*: Data are means (SD). Arterial stiffness data reported to 2 DP due to the meaningfulness of small changes. ^†^Significant time effect. cfPWV and sf‐SG *n* = 13; faPWV *n* = 14. Abbreviations: af‐SG, aortic‐femoral stiffness gradient; cfPWV, carotid to femoral pulse wave velocity; faPWV, femoral to ankle pulse wave velocity.

### 3.4 Lower limb blood pooling

There was a significant time × group interaction for calf circumference assessed pre‐ and post‐sitting (*P* < 0.001; β = −0.8, 95% CI: −1.1, −0.50). Simple effects found that calf circumference increased in the CON condition (*P* < 0.001; MD = 1.29, 95% CI: 1, 1.5 cm) and to a lesser extent in the ACT condition (*P* < 0.001; MD = 0.5, 95% CI: 0.3, 0.7 cm).

## 4 DISCUSSION

The aim of this study was to determine whether simple light physical activity interruptions can attenuate the expected detrimental responses to 2 h prolonged sitting on cardiovascular function in a population with established CHD. The main findings of this study were for CHD patients, light activity breaks every 30 min can attenuate the impact of prolonged sitting on SBP and cSBP, but it does not fully mitigate against DBP or arterial stiffness. Higher frequency or intensities of activity breaks may be required to preserve these.

### 4.1 Limitations and strengths

To fully contextualise the findings of this study, it is first important to acknowledge the strengths and limitations. Firstly, our research team has substantial expertise in conducting experimental research, with a demonstrated ability to recruit participants spanning both sexes and various disease statuses across the life‐course. Uniquely, although every attempt was made to recruit females with established CHD in the present study, none were successfully recruited. Because our sample included only older (mean 78.4 years, SD 6.24) Caucasian males, the generalisability of our findings is unfortunately restricted to this group. Our future work will seek to better understand the unique barriers female and ethnic CHD patients may face in becoming involved in research. Secondly, the lack of non‐CHD age‐matched controls in our research design prevents our understanding of whether altered responses to prolonged sitting or its interruption are due to ageing or pathologies unique to CHD; however, this was not our primary aim. Thirdly, we could not account for physical activity status nor cardiorespiratory fitness in our research design, which have been suggested as moderators of the cardiovascular response to sitting; however, our prior work in healthy adults suggests that these factors have no impact (Paterson et al., 2024). Lastly, we were not sufficiently powered to adjust the models for different medication usage, such as peripheral dilators, which may have impacted the responses to both CON and ACT conditions. Significant strengths of this study are that it is the first to evaluate the effectiveness of accepted prolonged sitting interruption strategies for preserving cardiovascular function in CHD patients and does so using an ecologically valid randomised crossover design with robust controls for effectors of BP and arterial stiffness.

### 4.2 Comparison to the literature

This is the first known study to assess the BP responses to prolonged uninterrupted and interrupted sitting in a population with CHD. Both SBP and cSBP significantly increased in response to uninterrupted sitting (CON), and this was attenuated when physical activity breaks were used every 30 min to interrupt sitting (ACT). Whilst our study is the first to show this response in a population with CHD, Dempsey et al. (2016) previously found increases of 10 mmHg in peripheral SBP over an 8 h period in those with type 2 diabetes. Compared to Dempsey et al. (2016) our data suggests that BP increases are greater in those with CHD over a shorter sitting duration (8 h vs. 2 h). This supports data from a recent meta‐analysis that found that the majority of the increase in BP, occurred within the first 2 h of sitting (Adams et al., 2024). In the current study, SBP and cSBP (Figure 1) significantly increased by 15 and 12 mmHg, respectively, in the CON condition, with a non‐significant increase of 4 and 3 mmHg, respectively, in the ACT condition. Given the exacerbated response to sitting in those with CHD over a shorter but more ecologically valid sitting time of 2 h, the benefits of interrupting sitting with physical activity breaks are favourable to CHD patients.

Whilst the physical activity interruptions used in this study were able to offset the effects of sitting‐induced increases in cSBP and SBP, they were unable to fully mitigate the increase seen in DBP and arterial stiffness assessed via cfPWV. This could in part be because of either the arterial remodelling caused by CHD or the effect of peripheral dilator medication (e.g., angiotensin‐converting enzyme inhibitors) some patients took. As this study was not sufficiently powered to covariate for medication usage, future research should look to determine the effects of pharmacological intervention on the BP response to uninterrupted and interrupted sitting. While there was no significant interaction for DBP, the magnitude of the response, as shown by the β‐values in (Table 2), was reduced with ACT, and so activity interruptions may still be favourable. It may be that DBP does not respond as favourably to intermittent exercise as SBP does. DBP is largely determined by arteriolar resistance and the Windkessel effect, and these are less affected by light physical activity (Pescatello et al., 2004; Pesova et al., 2023). It may be that more intense or regular physical activity interruptions are required to offset the uninterrupted sitting‐induced increases in DBP, as they may also be for markers of arterial stiffness (shown in Table 3). cfPWV and the af‐SG are markers of arterial stiffness which have been shown to independently predict (Vlachopoulos et al., 2010) and enhance the assessment of CVD risk (Stone et al., 2024). These are central to the sitting‐induced cardiovascular dysfunction model proposed by Stoner et al. (2021). As such, our novel finding that cfPWV is elevated (worsened) by almost 1 m/s after 2 h of uninterrupted sitting, and that this is not fully mitigated when light physical activity breaks are used to break up sitting every 30 min, is an important one. The magnitude of the cfPWV response in our study is larger than any previously reported data in young healthy people (0.3–0.6 m/s; Moinuddin et al., 2025), with the MD in the uninterrupted condition (0.97 m/s) almost reaching the clinically significant threshold of 1 m/s (Vlachopoulos et al., 2010). Previously, a 1 m/s chronic increase in cfPWV has been associated with a 14%, 15% and 15% increase in the future risk of cardiovascular events, mortality and all‐cause mortality, respectively (Vlachopoulos et al., 2010). The greater increase in cfPWV seen in this study (Table 3) may be due to the poor endothelial function associated with CHD in an already aged population, where the vessel is likely less elastic (Abrams, 1997). Impaired aortic endothelium loses its reservoir capacity and ability to buffer pulsatile flow, resulting in turbulent blood flow, reduced shear stress and diminished release of vasodilators such as nitric oxide, prostaglandin 2 and endothelial‐derived relaxing factors (Abrams, 1997; Stoner & Sabatier, 2012). Although we did not assess shear stress, prior work shows it declines after 3 h of sitting in young, healthy adults, alongside shear rate and oscillatory index (Credeur et al., 2019). Similar impairments are evident in CHD populations (Matsuzawa & Lerman, 2014), suggesting an additive effect on cfPWV that these light physical activity breaks cannot fully overcome. This contrasts with previous studies reporting improvements when sitting was interrupted (Fryer et al., 2023; Moinuddin et al., 2025), where increases in lower‐limb shear stress, blood flow and reduced venous pooling were proposed mechanisms. In CHD, however, endothelial dysfunction may blunt vasodilatory responses to movement (Abrams, 1997). Consistent with this, we observed a significant interaction in calf circumference, a proxy for venous pooling, yet 30 min activity breaks did not fully offset this increase either. Interestingly, this matches the significant decrease (time effect) seen for AIx and AIx@75, with the absence of any interaction effect (Table 1). The significant paradoxical decrease in AIx and AIx@75 has been well document (Credeur et al., 2019; Fryer et al., 2021, 2023), but given previous work has cited sitting induced lower limb venous pooling and the reduction in stroke volume as a potential mechanism for the common decrease in AIx and AIx@75 (Credeur et al., 2019), more work is needed to confirm this speculation. Currently, we propose that more frequent or higher‐intensity interruptions may be required to attenuate the vascular consequences of prolonged uninterrupted sitting, particularly for cfPWV. However, it should be noted that this study was powered based on BP and not cfPWV.

### 4.3 Implications

Our research extends the current literature by demonstrating that light physical activity interruptions appear sufficient to reduce BP in patients with established CHD. However, higher intensity, more frequent interruptions may be necessary to fully attenuate its impact – as evidenced by both DBP and arterial stiffness. The efficacy of using light physical activity interruptions to moderate the impact of sitting induced increases in BP is particularly important not just because of worse response to uninterrupted sitting, but due to its potential likelihood of its increased adoption compared to moderate to vigorous exercise. Whilst we did not assess potential barriers and facilitators to interrupting prolonged sitting with light physical activity, previous research suggests that at‐risk groups such as this are more likely to undertake light physical activity, opposed to moderate to vigorous activity, where they often feel at‐risk, stigmatised or incapable (Curran et al., 2024). Therefore, addressing population‐ and disease‐specific questions is critical to guide future recommendations, particularly where targeted sedentary behaviour interventions may be needed for groups with heightened sensitivity to sitting, or a diminished benefit from its interruption.

### 4.4 Conclusions

Sedentary behaviours such as prolonged uninterrupted sitting are an independent risk factor for CVD. Our study highlights that in a population with established CHD, prolonged uninterrupted sitting elevates BP and aortic stiffness. However, the effects on SBP and cSBP are attenuated when sitting is interrupted every 30 min with light physical activity. It may be that higher intensity, or more frequent physical activity interruptions are needed to fully attenuate the increases in DBP and cfPWV.

## AUTHOR CONTRIBUTIONS

Simon Fryer: study design, data collection, analyses, writing of the manuscript. Eve Scarle: study design, data collection, analyses, proofing of the manuscript. Louise Turner: study design, data collection, analyses, proofing of the manuscript. Arsalan Moinuddin: study design, data collection, analyses, proofing of the manuscript. James Faulkner: study design, analyses, proofing of the manuscript. Hayley Legg: analyses, proofing of the manuscript. Craig Paterson: study design, data collection, analyses, proofing of the manuscript. Keeron Stone: study design, analyses, writing of the manuscript. All authors have read and approved the final version of this manuscript and agree to be accountable for all aspects of the work in ensuring that questions related to the accuracy or integrity of any part of the work are appropriately investigated and resolved. All persons designated as authors qualify for authorship, and all those who qualify for authorship are listed.

## CONFLICT OF INTEREST

The authors declare no conflicts of interest.

## FUNDING INFORMATION

No funding was received for this work.

## Data Availability

The data that support the findings of this study are available from the corresponding author upon reasonable request.
